# Stability monitoring of some acetylcholinesterase reactivating drugs

**DOI:** 10.1186/1471-2210-11-S2-A52

**Published:** 2011-09-05

**Authors:** Nazila Ram, Péter Szegi, Kamil Kuča, Farzad Hashemi, Kornélia Tekes

**Affiliations:** 1 Department of Pharmacology and Pharmacotherapy, Semmelweis University, 1089 Budapest, Hungary; 2 Department of Pharmacodynamics, Semmelweis University, 1089 Budapest, Hungary; 3 Department of Toxicology, University of Defence, 500 01 Hradec Králové, Czech Republic

## Background

Widespread use of organophosphorous compounds (OPs) in agriculture and as nerve agents as well as a lack of clinically effective antidotes initiated the synthesis of new pyridinium bis-aldoximes (K-compounds) with high potency in reactivating acetylcholinesterase irreversibly inhibited by OPs [1,2]. We aimed to optimize an HPLC method sensitive enough to determine K-compounds from different biological matrices (blood, brain and cerebrospinal fluid) [3,4].

## Methods

Samples of biological origin needed proper clean-up. An RP-HPLC method using either UV and amperometric detector was used following separation on a Zorbax Rx-C18 octadecyl silica column with a mobile phase of phosphate buffer with 20% acetonitrile (pH 3.7). 1-Octane sulphonic acid sodium salt (OSA) was used as ion-pairing agent. Calculation of theoretical plate number, asymmetry of peaks, limit of quantitation (LOQ), lower limit of detection (LLOD) and determination of pH, temperature and OSA concentration dependence was done.

## Results

Elution characteristics of bis-pyridinium mono-aldoximes were depending on the OSA concentration, however, to a lesser extent than the bis-pyridinium bis-aldoximes. A double bond in the alkyl chain decreased the dependence from the ion-pairing agent concentration only to a minor extent. When the samples were kept at a pH under 1.5 a peak of degradation product was generated. The time course of degradation in an acidic milieu was calculated.

## Conclusions

Appropriate clean-up, optimal concentration of the ion-pairing agent and a well-selected mode of detection are the key factors for optimal determinations. We point out decomposition of pyridinium aldoximes at acidic pH.
